# Genome-Wide Association Mapping in *Arabidopsis* Identifies Previously Known Flowering Time and Pathogen Resistance Genes

**DOI:** 10.1371/journal.pgen.0010060

**Published:** 2005-11-11

**Authors:** María José Aranzana, Sung Kim, Keyan Zhao, Erica Bakker, Matthew Horton, Katrin Jakob, Clare Lister, John Molitor, Chikako Shindo, Chunlao Tang, Christopher Toomajian, Brian Traw, Honggang Zheng, Joy Bergelson, Caroline Dean, Paul Marjoram, Magnus Nordborg

**Affiliations:** 1 Molecular and Computational Biology, University of Southern California, Los Angeles, California, United States of America; 2 Department of Ecology and Evolution, University of Chicago, Chicago, Illinois, United States of America; 3 Cell and Developmental Biology, John Innes Centre, Norwich, United Kingdom; 4 Department of Preventive Medicine, Keck School of Medicine, University of Southern California, Los Angeles, California, United States of America; University of Wisconsin, United States of America

## Abstract

There is currently tremendous interest in the possibility of using genome-wide association mapping to identify genes responsible for natural variation, particularly for human disease susceptibility. The model plant *Arabidopsis thaliana* is in many ways an ideal candidate for such studies, because it is a highly selfing hermaphrodite. As a result, the species largely exists as a collection of naturally occurring inbred lines, or accessions, which can be genotyped once and phenotyped repeatedly. Furthermore, linkage disequilibrium in such a species will be much more extensive than in a comparable outcrossing species. We tested the feasibility of genome-wide association mapping in *A. thaliana* by searching for associations with flowering time and pathogen resistance in a sample of 95 accessions for which genome-wide polymorphism data were available. In spite of an extremely high rate of false positives due to population structure, we were able to identify known major genes for all phenotypes tested, thus demonstrating the potential of genome-wide association mapping in *A. thaliana* and other species with similar patterns of variation. The rate of false positives differed strongly between traits, with more clinal traits showing the highest rate. However, the false positive rates were always substantial regardless of the trait, highlighting the necessity of an appropriate genomic control in association studies.

## Synopsis

There is currently tremendous interest in using association mapping to find the genes responsible for natural variation, particularly for human disease. In association mapping, researchers seek to identify regions of the genome where individuals that are phenotypically similar (for example, they all have the same disease) are also unusually closely related. A potentially serious problem is that spurious correlations may arise if the population is structured so that members of a subgroup tend to be much more closely related. Because few genome-wide association studies have been carried out, it is not yet known how important this problem will be in practice.

In one of the first genome-wide association studies to date, this paper considers the model plant *Arabidopsis thaliana.* A very large number of spurious genotype–phenotype correlations are found, especially for traits that vary geographically. For example, plants from northern latitudes flower later; however, in addition to sharing genetic variants that make them flower late, they also tend to share variants across the genome, making it difficult to determine which genes are responsible for flowering. This notwithstanding, several previously known genes were successfully identified in this study, and the researchers are optimistic about the prospects for association mapping in this species.

## Introduction

One of the main challenges of modern biology is achieving a better understanding of the molecular genetic basis for naturally occurring phenotypic variation. Primarily because of rapidly decreasing genotyping costs, genome-wide association mapping (also known as linkage disequilibrium mapping) has emerged as a very promising tool for accomplishing this. The basic idea is simple: rather than looking for marker–trait associations in a population with known relationships (such as the members of a pedigree, or the offspring of an experimental cross), we look for associations in the general population of “unrelated” individuals [1]. Because unrelated individuals are, of course, always related at some distance, phenotypically similar individuals may be similar because they share alleles inherited identical by descent, alleles that will be surrounded by short ancestral marker haplotypes that can be identified in genome-wide scans. Association mapping has two main advantages over traditional linkage mapping methods. First, the fact that no pedigrees or crosses are required often makes it easier to collect data. Second, because the extent of haplotype sharing between unrelated individuals reflects the action of recombination over very large numbers of generations, association mapping has several orders of magnitude higher resolution than linkage mapping.

The drawbacks of association mapping stem from the fact that it is not a controlled experiment. Power is unpredictable, partly because the decay of linkage disequilibrium is noisy, and partly because the genetic architecture of the trait is unknown (the latter is always a problem in mapping complex traits, but it is likely to be worse in association mapping because genetic heterogeneity is not limited by a small number of founders) [1–3]. The false positive rate is similarly difficult to predict: it is well known that population structure can cause strong spurious correlations [4]. The severity of these problems is not known, because few (if any) genome-wide association studies have been carried out to date.

Highly selfing organisms, like *Arabidopsis thaliana,* are ideal candidates for association mapping. First, they largely exist as collections of naturally occurring inbred accessions, which can be genotyped once and phenotyped repeatedly, for the same phenotype (to reduce environmental noise) or different phenotypes (allowing “in silico mapping” [5]). Second, inbreeding results in a pattern of polymorphism characterized by extensive haplotype structure, which should be well suited for association mapping [6].

Preliminary studies indicated that linkage disequilibrium in *A. thaliana* decayed over 50–250 kb [7]. Based on these results, a genome-wide polymorphism survey in which short (500–600 bp) fragments were resequenced approximately every 100 kb in 95 individuals was carried out. Analysis of these data resulted in two findings of direct relevance to association mapping [8]. First, linkage disequilibrium appears to decay faster than predicted, within 50 kb. This means that the available polymorphism data are not dense enough for a genome-wide association study. Second, *A. thaliana* exhibits substantial population structure. This means that the sample is less ideal for association mapping for the reasons alluded to above.

In spite of these problems, we have used the data to investigate the feasibility of genome-wide association mapping in *A. thaliana*. We considered four phenotypes for which major loci are known (the vernalization response locus *FRI* [9] and the three pathogen resistance loci, *Rpm1, Rps5,* and *Rps2* [10–12]), and asked whether these loci could have been identified using genome-wide association mapping given a small, heavily structured sample such as the one available to us. We found that, in spite of an extremely high false positive rate, we were able to identify all of them, thus demonstrating the potential of genome-wide association studies in *A. thaliana,* and other species with similar patterns of variation.

## Results

### Genome-Wide Associations and the False Positive Rate

**Figure 1:**
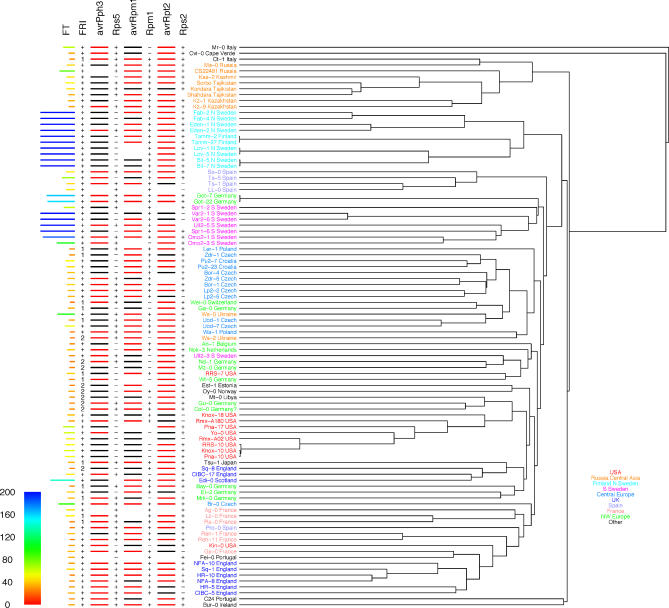
Summary of the Data Used in the Study The columns on the left give the genotype and associated phenotype for four loci, for each of the 95 accessions. The four loci are the flowering time locus *FRI* (+, wild-type; 1, *Ler* null allele; 2, *Col* null allele [9]), for which the associated phenotype is flowering time in long-day conditions without vernalization (late flowering is indicated by height and color of bar), and the three pathogen resistance loci *Rps5*, *Rpm1*, and *Rps2* (+, wild-type; −, null allele [10,11,12]), for which the associated phenotypes are hypersensitive response to the appropriate bacterial *avr* gene (red indicates resistance, black indicates susceptibility, and missing data are indicated by missing bar). The tree on the right illustrates the genetic relationships between the accessions [8]. It is clear that phenotypes and genotypes are correlated, genome-wide.

**Figure 2:**
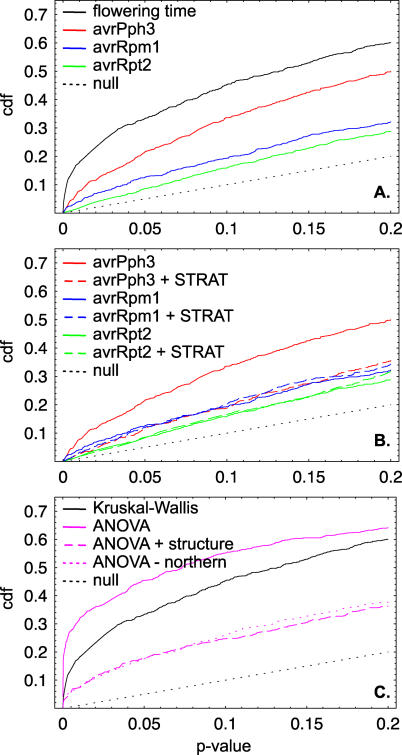
The Genome-Wide Distribution of *p*-Values under Different Scenarios (A) Cumulative distribution of *p-*values for association tests across approximately 850 loci. The sequenced haplotypes at each locus were treated as alleles (after eliminating singleton polymorphisms), and the significance of genotype–phenotype associations was tested using Kruskal–Wallis tests in the case of flowering time (a continuous trait), and using χ2 tests in the case of resistance (a binary trait). Under the null hypothesis of no association, the cumulative distribution should be a straight line: the observed distributions are all heavily skewed towards zero. (B) The cumulative distribution of *p-*values for association with pathogen resistance, with and without correction for population structure using the program STRAT [13]. The false positive rate is decreased for avrPph3, but is unaffected for the other two phenotypes. (C) The cumulative distribution of *p-*values for association with flowering time, with and without correction for population structure. ANOVA was used instead of the nonparametric Kruskal–Wallis test to make it possible to use population structure as cofactor (cf. [14]). The distribution for ANOVA with accessions from Finland and northern Sweden removed is also shown (“ANOVA − northern”). The false positive rate is decreased using both approaches.

**Figure 3:**
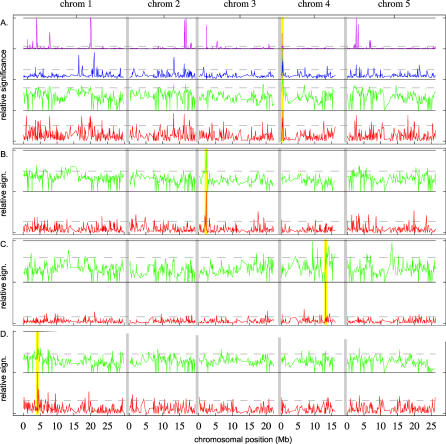
Genome-Wide Scans for Association with Flowering Time and Pathogen Resistance For flowering time (A), four different statistical methods were used (described in Materials and Methods): Voronoi focusing on “late” alleles (magenta line), Voronoi focusing on “early” alleles (blue line), CLASS (green line), and fragment-based Kruskal–Wallis tests (red line; see also Figure 2). For pathogen resistance (avrRpm1 [B], avrRpt2 [C], and avrPph3 [D]), only the last two tests were used. Higher peaks indicate stronger association (the *y*-axes are proportional to the negative log *p-*values, but have been normalized to the highest value within each test). The dotted lines correspond to the 95% percentile and are mainly intended to facilitate comparison between figures. Yellow vertical lines indicate the positions of the appropriate candidate loci. Peaks occur at these loci for all methods, but are otherwise distributed throughout the genome.

The data used in this study are summarized in Figure 1, which shows genotype and associated phenotype for four genes, for each of the 95 accessions, plotted against a tree representing the genome-wide relationships among the accessions (from [8]). The tree illustrates that accessions whose origins are geographically close tend to be more closely related, and it is clear by inspection that the phenotypes are not randomly distributed with respect to this tree. Flowering time was particularly strongly correlated with geographic origins, as would be expected for a trait that is likely to be under clinal selection. It follows that the standard null hypothesis in association mapping, independence between marker genotypes and traits, is false in a genome-wide sense. In other words, we should expect an elevated false positive rate, and this is precisely what we found. As illustrated in Figure 2, the distribution of *p-*values across the genome was heavily skewed towards zero, with flowering time showing the strongest deviation from the null expectation. To give some idea of the magnitude of the deviation, a naive application of a Kruskal–Wallis nonparametric test of association between flowering time and each of the approximately 850 sequenced loci (treating haplotypes as alleles) yielded 7% significant tests at the (nominal) 0.1% level, 18% significant tests at the 1% level, and 33% at the 5% level. The (nominally) significantly associated loci were distributed throughout the genome (Figure 3) and are clearly not all true positives. Indeed, given that we expect our study to have low power (due to both insufficient marker density and genetic heterogeneity), it is possible that none, except the previously known loci, are true positives.

We attempted to decrease the false positive rate by taking population structure into account using so-called structured association, in which one uses genome-wide markers to infer population structure, and then carries out association tests conditional on the inferred structure [13,14]. For the pathogen resistance phenotypes, structured association reduced, but did not eliminate, the elevated positive rate for the most biased of the phenotypes (response to avrPph3); it had no effect on the other two rates (see Figure 2B). Similarly, the false positive rate for flowering time was strongly reduced, but remained extremely elevated relative to null expectations (Figure 2C). It is clear from Figure 1 that, at least for flowering time, much of the elevated false positive rate is due to the Swedish and Finnish accessions, which are genetically distinct and phenotypically extreme. Indeed, removing these accessions from the analysis reduced the false positive rate as much as using structured association (Figure 2C).

### Mapping of Known Loci for Flowering Time and Pathogen Resistance

**Figure 4:**
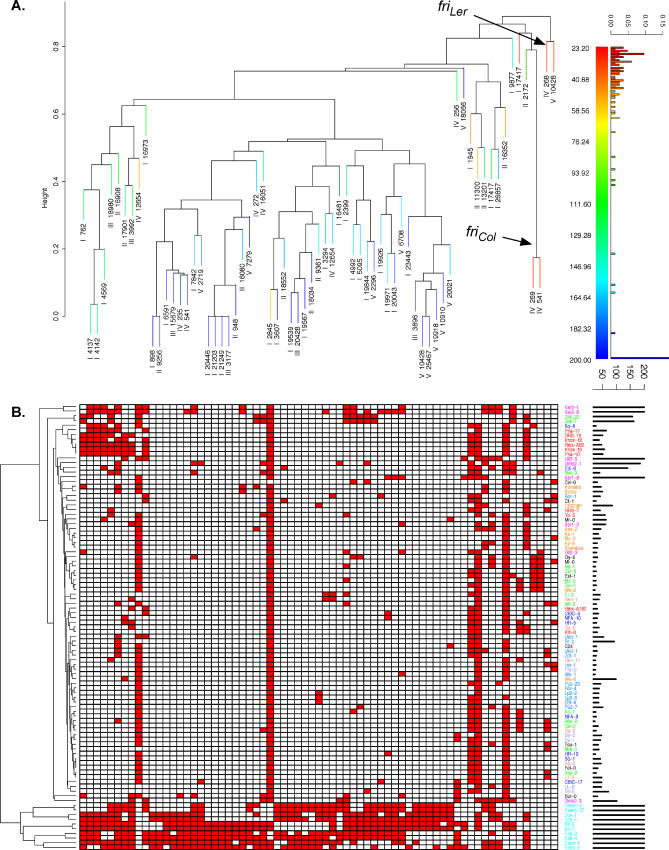
Haplotypes Significantly Associated with Flowering Time Clustered by Haplotype Membership To help determine which associations were real and which were due to population structure, the most significantly associated haplotypes (based on fragment-wise Kruskal–Wallis; see Materials and Methods) were clustered based on similarity in the list of accessions that carry each haplotype. (A) The tree shows the resulting cluster with tips colored according to average flowering time among the accessions that carry the haplotype corresponding to each tip (the scale is given on the right along with a histogram showing the distribution of flowering time across the 95 accessions). (B) The matrix shows the membership list for each haplotype. Each column corresponds to the haplotype (tip) in the tree above it; accessions highlighted in red carry the haplotype significantly associated with flowering time. The tree thus illustrates the clustering of the columns of the matrix: clustering was done based on pairwise distance as measured by the absolute value of the correlation in membership between columns. Phenotypes of the accessions are given on the right, and the rows of the matrix (i.e., the accessions) have been clustered based on pairwise Hamming distance. It is evident that most of the significant haplotypes, regardless of position in the genome, share similar membership lists that include the accessions from Finland and northern Sweden. On the other hand, the clusters corresponding to the known major alleles of *FRI* are unique, indicating that these are indeed true positives.

In spite of the high false positive rate, the four known loci were detectable in genome-wide scans (Figure 3). For the three pathogen resistance phenotypes the strongest association was found inside the appropriate *R* gene regardless of association method used. For flowering time, strong associations were evident in multiple locations throughout the genome, but associations in the *FRI* region were invariably among the ten most significant. Furthermore, *FRI* could readily be distinguished as true positive by clustering associations on the basis of which accessions were part of each association. Our rationale was that false positives due to population structure are expected to reoccur across the genome. This is precisely what we saw. Our haplotype-based association statistics identified loci for which clusters of phenotypically similar accessions exhibited excessive haplotype sharing. Figure 4 shows the result of clustering these clusters based on similarity in membership. We found that the vast majority of all significant associations were due to haplotype sharing among accessions from Finland and northern Sweden, sometimes with North American accessions also included. This type of association is thus found across the genome, and while nominally significant, is not significant in a genomic sense. Note that this does not mean that all these associations are false positives, but it does mean that most of them are. The very late flowering phenotype of the Finnish and northern Swedish accessions does have a genetic basis: we have identified a list of candidates, but we have no way of telling which (if any) of them is true.

Figure 4 also identifies clusters with the property of being unique across the genome. In a hierarchical clustering, these would represent the deepest nodes because they are dissimilar from other clusters. Among the small number of “unique” clusters we identified one that corresponds to haplotype sharing among accessions carrying the *Ler* loss-of-function allele at *FRI,* and one that corresponds to the *Col* loss-of-function allele at the same locus [9]. These associations thus have the property that, in addition to being (nominally) significant, they are not found repeatedly across the genome. They are therefore more likely to be true positives.

**Figure 5:**
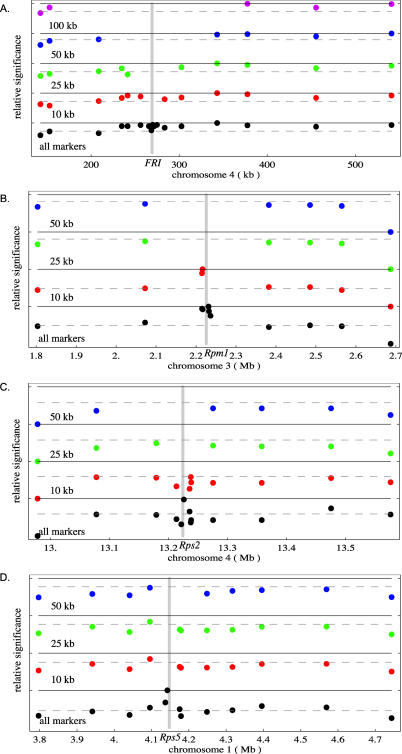
The Strength of Association (Using CLASS) around the Four Candidate Loci for Various Marker Densities For each locus (*FRI* [A], *Rpm1* [B], *Rps2* [C], and *Rps5* [D]), the bottom panel shows the pattern of association using all available fragment markers around the locus (the position of which is given by a grey vertical line), and the panels above show the effect of successively reducing the marker density so that no markers are within 10, 25, 50, and 100 kb (*FRI* only) of the causative polymorphisms. The dotted grey line represents the 95th percentile of all associations across the genome. Because we used an association statistic that utilizes the pattern of haplotype sharing across multiple fragments, the relative significance of any particular fragment may change depending on the presence or absence of other fragments. The *FRI* region (A) remains strongly associated with flowering time even for the lowest marker density, while the signal of association around the *R* genes (B–D) disappears as one goes from 10- to 25-kb spacing.

The above analyses were intended to demonstrate that the signal of genotype–phenotype association for these four major loci would have been sufficient for genome-wide association mapping even in the small, heavily structured sample used by Nordborg et al. [8]. We have not addressed the other main aspect of power in association mapping, namely, the extent of linkage disequilibrium and what it implies about the marker density required for genome-wide scans. As mentioned in the Introduction, the marker density in the data of Nordborg et al. [8]—one resequenced fragment every 100 kb—is insufficient to cover the genome. The results above were based on denser marker coverage around the four loci, including markers within each target gene. As it turns out, we would have detected *FRI* and *Rpm1* without adding additional markers, the former because (as we shall see below) the original marker coverage was sufficient to detect *FRI,* the latter because of luck. However, the denser marker coverage around all four loci allowed us to determine the required marker density by thinning the markers and noting when the signal disappeared. Figure 5 shows the result of successively eliminating resequenced fragments so that no markers were within 10, 25, 50, and 100 kb of the target locus. The difference between *FRI* and the three *R* genes is striking: while the former was readily picked up with the lowest marker density (corresponding to the density in the genome-wide data), the latter were only picked up with 10-kb spacing. When markers within 25 kb were eliminated, the association signal for the *R* genes was typically lost.

## Discussion

### Genome-Wide Association Mapping and Population Structure

Our results present a striking demonstration of the potential effect of population structure in causing an elevated false positive rate in association mapping. As genome-wide association studies in humans are becoming increasingly feasible, the seriousness of this problem has been the subject of considerable debate [15–19]. In this context, our study is roughly equivalent to a genome-wide scan for association with skin color using a world-wide sample of humans. Most human association mapping studies are likely to be case–control studies, which, given a judiciously chosen control, should be less prone to false positives [17].

Nonetheless, more studies like ours are likely to be carried out, in humans as well as in other organisms, and it seems likely that population structure will then be a problem. The extent of the problem will of course depend on the extent to which the sample is structured, but it will also depend on the phenotype. Traits that are strongly correlated with population structure will display a more highly elevated rate of false positives. In the present case, flowering time, which is likely involved in local adaptation [20,21], shows a more highly elevated rate than pathogen resistance, variation for which appears to be maintained by frequency-dependent balancing selection [10–12]. It should be noted, however, that differences between the resistance phenotypes were also found: the false positive rate for avrPph3 is more highly elevated than for the other resistance-related rates (see Figure 2). Why this should be the case is not clear, but might tell us something about the ecology of the pathogens responsible for maintaining polymorphism at these loci.

Several methods for dealing with false positives due to population structure have been proposed. The best known are “genomic control” [22] and “structured association” [13]. We found that structured association based on the approach of Pritchard et al. [13] and Thornsberry et al. [14] did not successfully correct the elevated false positive rate in our sample. This should not be surprising. The model underlying the approach of Pritchard et al. [13] is one of admixture between a small number of homogeneous, randomly mating populations. While this may be a reasonable approximation for many human samples, it is clearly not valid for our sample of *A. thaliana,* which shows all signs of isolation by distance [8].

Genomic control [22] is an alternative approach in which genome-wide markers are used to estimate the effect of population structure on association statistics and correct these statistics to achieve valid significance levels. We did not try this approach for several reasons. First, it, too, is based on a simple model of population structure. Second, the approach has only been developed for relatively simple contingency table statistics, and it is not clear how it should be implemented for the haplotype-based methods used here. Third, it is clear from our *FRI* results that genomic control would lack power. Association with *FRI* is not necessarily stronger than the false positives due to structure, and any approach that eliminated the latter based on the strength of association would also eliminate the former. In contrast, Figure 4 suggests that methods that simultaneously infer the structure and the associations should be able to separate true from false positives.

It is clear that more work is needed in this area. Indeed, given the difficulty of modeling population histories, it may be fruitful to abandon the notion of “population structure” (with its implication that unstructured populations actually exist), and instead view all population samples as members of a gigantic, unknowable pedigree. Models appropriate for handling such data have been developed in the animal breeding community [23], and can be extended to genome-wide association mapping [24,25].

### The Prospect for Genome-Wide Association Mapping in *A. thaliana*

We have demonstrated that *FRI, Rpm1, Rps2,* and *Rps5* could have been detected using genome-wide association mapping even in the small and heavily structured sample used by Nordborg et al. [8]. It should be emphasized that these are genes of major effect: the two loss-of-function alleles at *FRI* account for 13% of the variation in flowering time in our study, and correlation between being susceptible and carrying the known susceptibility allele is 0.66, 0.77, and 0.62 for *Rpm1, Rps2,* and *Rps5,* respectively. To map genes of more subtle effect, a much larger sample is surely needed. Furthermore, since power in association mapping is determined both by the effects of alleles and by their frequencies [3,26], the structure of the sample matters greatly. In addition to elevating the false positive rate, the presence of population structure may increase genetic heterogeneity—avoiding this problem is one of the main arguments for the use of population isolates in human genetics [27]. Whether genetic heterogeneity is a problem or not depends on the genetic architecture of the trait, which is of course unknown a priori.

In addition to a different sample, it is clear that a denser marker map than the one generated by Nordborg et al. [8] is needed. Although we were able to map *FRI* using 100-kb marker spacing, it is now clear that linkage disequilibrium around this gene is unusually extensive, probably because of a combination of local adaptation and recent selective sweeps (as was suggested by earlier studies [7]). On the other hand, the extent of linkage disequilibrium surrounding the *R* genes is likely to be smaller than usual because variation at these loci is due to ancient polymorphism maintained by balancing selection [10,11]. The observation that we can map these genes using linkage disequilibrium with markers 10 kb away suggests that a marker spacing of roughly 20 kb (which guarantees at least one marker within 10 kb of a causative polymorphism) would provide reasonable power. This implies that on the order of 6,000 single nucleotide polymorphisms (SNPs) chosen to be maximally informative about the local haplotype structure (so-called tag-SNPs [28,29]) might be sufficient for genome-wide association mapping in *A. thaliana*. Needless to say, the marker spacing required will vary across the genome depending on the local haplotype structure, and also depends on the sample. Further studies to investigate the required density are underway.

## Materials and Methods

### Plant material.

The accessions used are described in [8].

### Sequencing and genotyping.

We used the resequencing data of Nordborg et al. [8], plus additional fragments resequenced around the four loci. Genotyping for the loss-of-function deletion alleles at *FRI, Rpm1,* and *Rps5* was done using PCR assays as previously described [10,11,21]. Genotyping at *Rps2* (not a deletion polymorphism) was done by sequencing the entire leucine-rich repeat region and comparing the results with those of [12]. All data are available as Datasets S1 and S2.

### Measuring flowering time.

Flowering time was measured in days using plants grown under long-day conditions (16 h light, 8 h dark) at a constant temperature of 18 °C. Measurements were generally taken for six plants per accession, and the average used in the analysis. The experiment was stopped at 200 d, and accessions that had not flowered at that point were assigned a value of 200. The flowering time data are available as Dataset S1.

### Measuring pathogen resistance.

Seedlings of each accession were germinated in flats containing a 1:1 mixture of Premier Pro-Mix and MetroMix (Premier Horticulture, Red Hill, Pennsylvania, United States). Flats were first placed at 4 °C for 7 d to promote germination, then placed in a growth room at 20 °C with short-day lighting (12 h light, 12 h dark). On the 23rd day of growth, two leaves per plant were inoculated with 0.1 ml of 10^8^ cfu/ml bacteria in 10 mM MgSO_4_ buffer using a blunt-tipped syringe [30]. Leaf collapse was scored at 20 h and again at 24 h after inoculation. A positive score at either time point was deemed a hypersensitive response. The four *avr* genes were tested using the following transformed strains of *Pseudomonas syringae:* Pst DC3000::avrPphB [31], Pst DC3000::avrRpm1 [32], Pst DC3000::avrB (from J. Greenberg, University of Chicago), and Pst DC3000::avrRpt2 [33]. As a negative control, *P. syringae* DC3000 without the *avr* genes was also tested [33]. Each of the five strains was tested in a separate experiment consisting of six replicates of each of the 95 accessions, planted two per cell, for a total of 576 plants and six flats in each test. Accessions were considered to exhibit a hypersensitive response if at least eight of the 12 replicate leaves exhibited collapse. Accessions were considered to lack the hypersensitive response if at least eight of the 12 replicate leaves exhibited no leaf collapse. Accessions that exhibited ambiguous responses to a strain were excluded from further analysis. The negative control strain, *P. syringae* DC3000 without the added *avr* genes, caused no hypersensitive response in any of the lines. Results for avrPphB were almost identical to those for avrRpm1, and are not shown. The resistance data are available as Dataset S1.

### Association mapping methods.

There has been considerable debate over how much power is gained by using haplotype-based instead of single SNP methods. In organisms where linkage disequilibrium decays rapidly (e.g., *Drosophila melanogaster* [26]), or where haplotypes have to be inferred (e.g., humans [34,35]), this is indeed a relevant question. In the present case, the polymorphism data come in the form of short haplotypes within which linkage disequilibrium is nearly complete, and it is thus natural to utilize haplotype-based methods. Indeed, we have found that methods incorporating longer-range disequilibrium sometimes perform substantially better [40]. We utilized three different methods here.

### Single-fragment haplotypes.

After removing singleton polymorphisms, each resequenced fragment was treated as a multi-allelic marker locus with haplotypes corresponding to alleles. Haplotypes with frequency lower than 5% were grouped. Phenotypic associations were then tested using either a Kruskal–Wallis test in the case of flowering time (a continuous trait), or χ^2^ tests in the case of resistance (a binary trait).

### CLASS (cladistic association).

We developed a simple clustering method similar in spirit to what has been proposed by several other researchers [36–38]. For each resequenced fragment, we first generated a similarity matrix using the extent of pairwise haplotype sharing between all pairs of accessions. We then clustered the accessions using a standard hierarchical clustering algorithm (we used neighbor joining), and heuristically searched for clades of accessions that were strongly associated with the phenotype (using either Kruskal–Wallis or χ^2^ tests to evaluate the strength of association). Our algorithm found clades using the following steps. (1) Search all clades and choose the one that gives the lowest *p-*value in a test with one degree of freedom. (2) Search the tree obtained by removing this clade for the clade that gives the lowest *p-*value in a test with three factors (and two degrees of freedom): the target clade, the clade identified in the previous step, and the remaining individuals. We repeated step 2, increasing the degrees of freedom by one each step, until the *p-*values no longer decreased.

### Voronoi.

We utilized a slightly modified version of the spatial clustering algorithm described elsewhere [39] and that has previously been used to fine-map *FRI* [40]. To summarize, each haplotype cluster searched by Voronoi contains a prototypic haplotype to which all observed haplotypes are compared, with respect to a starting location, or center. The simple similarity measure used to compare the two haplotypes is the calculated shared length identical by state originating from the center. Standard Markov chain Monte Carlo techniques were used to identify parameters such as haplotype risks for each cluster, which could then associate a haplotype cluster to an observed phenotype.

We deviated from the original version of this algorithm by assigning haplotypes to a specific cluster in a probabilistic way rather than a deterministic fashion. At any given step of the Markov chain Monte Carlo algorithm, a randomly observed haplotype was selected as the prototypic haplotype. We then assigned haplotype *h_i_* to cluster *c_n_* according to the following probability:


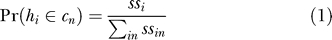


where *ss_in_* is the normalized shared length between the *h_i_* haplotype and 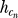 cluster center haplotype. *ss_in_* is the ratio of the observed and the mean shared length at *x_c_,* where *x_c_* is the putative functional mutation location in cluster *c*.

Furthermore, rather than using the Bayes factor as a summary statistic, we used the posterior likelihood as our final statistic. We constructed the 95% confidence interval of the likelihood for each haplotype and considered a haplotype to be significant if the confidence interval did not contain zero. This procedure also allowed the distinction between positive and negative effects. For those significant haplotypes, if the confidence interval was above zero, we concluded a positive association to late flowering; otherwise, the haplotype was negatively associated with early flowering. The posterior likelihood distribution of the functional mutation associated with the significant haplotypes gave likelihood for both positive and negative effects.

### Significance thresholds.

To generate the clustering in Figure 4, the 75 most significant fragments were selected, and, from among these, all haplotype clusters with a Bonferroni-corrected *p-*value less than 0.005 were selected. Note that the *p-*value for a fragment reflects all haplotypes observed for that fragment (the number of categories in the Kruskal–Wallis tests equals the number of haplotypes), whereas the *p-*value for a particular haplotype reflects the contribution of that haplotype only (two categories). These thresholds were chosen to yield an interpretable figure.

### Correcting for population structure.

We attempted to decrease the false positive rate due to population structure using structured association, in which one looks for associations conditional on inferred population structure [13]. We used the population structure estimate from the program STRUCTURE [41], with *K* = 8 clusters, generated as described in [8].

For the binary pathogen resistance phenotypes, association analysis was then carried out using the program STRAT [13]. However, since STRAT only works with binary data, it could not be used with the quantitative flowering time phenotype. Thornsberry et al. [14] extended the structured association approach to quantitative phenotypes, but their method is restricted to binary (SNP) genotypes, and cannot be used with the haplotype data available to us. Instead, we used a simple modification, in which the cluster assignment produced by STRUCTURE (the *Q* matrix) was used as a cofactor in a standard ANOVA. Basically, we carried out a likelihood ratio test of two models: *H*_0_ was FT ~ *Q* and *H*_1_ was FT ~ as.factor(marker genotype) + *Q*. The *p*-values were based on the χ^2^ distribution of the likelihood ratio test statistic.

## Supporting Information

Dataset S1 Genomic Alignments (1.3 MB ZIP)

Dataset S2 Genotypes and Phenotypes (3 KB CSV)

## Abbreviations

SNP: single nucleotide polymorphism
